# Relationship of caffeine regimen with osteopenia of prematurity in preterm neonates: a cohort retrospective study

**DOI:** 10.1186/s12887-022-03493-x

**Published:** 2022-07-21

**Authors:** Manoj Kumar, Amin Ali, Muhammad Azeem Khan, Sadia Sohail, Syed Muzafar Saleem, Midhat Khan, Fizzah Naz, Wasif Ahmed Khan, Muhammad Sohail Salat, Kashif Hussain, Gul Ambreen

**Affiliations:** 1 Department of Pharmacy, Aga Khan University Hospital, Karachi, Pakistan; 2 Department of Paediatrics & Child Health, Aga Khan University, Karachi, Pakistan; 3 Department of Neonatology & Paediatrics, Dow University of Health Sciences, Karachi, Pakistan; 4 Department of Neonatology & Paediatrics, Medicare Hospital, Karachi, Pakistan; 5 Department of Paediatrics, Fatimiyah Hospital Paediatrics, Karachi, Pakistan

**Keywords:** Caffeine, Apnea of prematurity, Metabolic bone disease, Osteopenia of prematurity, Preterm neonates, NICU

## Abstract

**Background:**

Caffeine is a routinely prescribed pharmacological active compound in neonatal intensive care units (NICU) for treating apnea of prematurity (AOP), which also decreases the risk of bronchopulmonary dysplasia and cerebral palsy in neonates. Caffeine-induced excessive calcium loss can promote the development of metabolic bone disease (MBD) in preterm neonates. This study aimed to evaluate the effect of the caffeine regimen on the development of osteopenia of prematurity (OOP), using serum alkaline phosphatase (serum-ALP) concentrations as a surrogate marker at the 4^th^ week of life.

**Methods:**

This retrospective cohort study was conducted including neonates of < 32 weeks gestational age (GA) and birth weight < 1500 g, admitted to NICU from April-2017 to December-2018 and received caffeine therapy till 28 days of life for AOP. Based on serum-ALP levels, formed the high and low-ALP groups. Neonatal characteristics, caffeine regimen, risk factors for OOP, including duration of parenteral nutrition (PN), exposure to medicines associated with MBD, and intake of essential vitamins and minerals, were compared in both groups. Predictors of OOP were analyzed through logistic regression.

**Results:**

From the total of 268 participants, 52 (19%) developed OOP, mostly female (61.5%). In the high ALP group, the serum-ALP levels were significantly higher than in the low-ALP group (725.0 ± 143.8 vs 273.6 ± 55.0 units/L, *p* < 0.001). The high-ALP group received significantly (*p* < 0.001) higher daily and cumulative caffeine doses and were associated with a higher likelihood of developing OOP in this study cohort [cumulative dose (mg) (AOR = 1.082 95% CI 1.011 to 1.157) and daily dose (mg/kg/day) (AOR = 2.892 95% CI 1.392 to 6.007)]. Smaller GA was found directly related to OOP. Among the other medical risk factors, phosphorus intake was significantly low in the high-ALP group. No, significant relationship between duration of PN and use of steroids and diuretics, and intake of vitamins and minerals were identified.

**Conclusion:**

The daily and cumulative doses of caffeine and smaller GA are associated with the development of OOP in this study cohort. Clinical randomized control studies are needed to validate the outcomes and determine the range of safest and most effective caffeine doses for treating AOP in preterm neonates.

**Supplementary Information:**

The online version contains supplementary material available at 10.1186/s12887-022-03493-x.

## Background

Most bone mineralization takes place in the 3^rd^ trimester of pregnancy with a peak at the 34^th^ week of gestation [1] Thus, preterm infants are born with less bone mineral density (BMD) [2]. In addition, bone resorption (BR) is significantly related to the development of osteopenia of prematurity (OOP) and is increased by the physiological adaptation of bone to extra-uterine life [3]. Therefore, in preterm babies, the process of BR starts earlier than in term babies. Every year more than 15 million babies are born prematurely worldwide, which is about 10% of the births [4]. The severity and incidence of OOP are inversely related to gestational age (GA) and birth weight (BW) more premature and lower the BW more the incidence and severity of OOP [5].

Caffeine is routinely prescribed pharmacological active compound in neonatal intensive care units (NICU) for treating apnea of prematurity (AOP) and to facilitate extubation [6, 7]. For treating AOP the recommended loading dose of caffeine citrate is 20 mg/kg, followed by a maintenance dose of 5 to 10 mg/kg/day [8]. In addition, caffeine increases minute ventilation, improves carbon dioxide sensitivity, and prevents diaphragmatic fatigue by enhancing its activity [9]. Caffeine therapy for AOP has also been shown to reduce the rate of bronchopulmonary dysplasia and improves the rate of survival of very low birth weight (VLBW) infants without neurodevelopmental disability at 18–21 months [10, 11]. The serum half-life of caffeine ranges from 40–230 h, decreasing with advancing postmenstrual age until 60 weeks. After oral administration, the peak serum levels are achieved after 30–120 min and about 85% of the drug is excreted unchanged in urine [12].

Caffeine metabolizing hepatic enzymes mature with advancing GA therefore its clearance is distinctly lower with the higher volume of distribution in preterm infants than in term infants [13, 14]. Thus, preterm neonates are at higher risk of experiencing adverse renal effects such as diuresis and natriuresis [9, 15], which leads to negative calcium (Ca) balance [16]. In preterm neonates, this excessive urinary Ca-loss can promote the development of metabolic bone disease (MBD) i.e. OOP [17–19]. Other than caffeine there are medicinal risk factors for developing OOP like prolonged use of parenteral nutrition (PN) (> 3–4 weeks), drugs, including loop diuretics, corticosteroids, antiepileptic, and immobilization [17–19].

In the clinical setting, serum-alkaline phosphatase (Serum-ALP), serum phosphorous (Serum-P), and serum-Ca have been commonly used for MBD screening in preterm neonates due to their simplicity and ease of measurement [20, 21]. Because elevation in Serum-ALP mostly precedes radiological changes and prompts an additional screening for the confirmation of the diagnosis of OOP. However, the detection of early-onset and mild to moderate forms of MBD may not possible through radiological screening as radiographical changes are not evident until bone mineralization is decreased by ≥ 20% [18]. Serum-ALP > 500 units/L has a sensitivity of 100% and specificity of 81% to detect radiologic OOP [20]. In addition, serum-*P* levels < 5.6 mg/dL (< 1.8 mmol/L) are reported to have a greater risk for MBD and development of OOP with diagnostic sensitivity and specificity of 70% and 100%, respectively [22].

The caffeine-induced renal Ca-excretion and lower bone mineral density (BMD) prompted the researchers for this retrospective assessment of the association between caffeine regimen and risk of OOP development, using serum-ALP concentrations as a surrogate marker for OOP or MBD. The primary outcome measures of this study were to compare the neonatal characteristics and caffeine regimen between the high ALP and low ALP groups, including maximum daily doses and cumulative doses. Secondary outcomes included a) comparison of other risk factors for OOP, including duration of PN, exposure to medicines associated with OOP, and intake of essential vitamins and minerals through enteral and parenteral. b) The probability of OOP by GA after establishing the relationship between GA and OOP.

## Methods

### Study design, setting, and population

This retrospective study was conducted in Aga khan university hospital (AKUH), a tertiary care setting in Karachi, Pakistan. The AKUH has the facility of 24 bedded multispecialty tertiary care NICU. About 1200 neonates are admitted annually with the influx of very preterm high-risk newborns from all over the country. All the preterm neonates were included in the initial cohort who were admitted to NICU and had GA < 32 weeks and BW < 1500 g and were administered caffeine for treating AOP during the study period (April 2017-December 2018). Neonates were identified from the hospital electronic database. All the neonates with congenital defects of the endocrine system, and renal or bone mineralization/formation were excluded. Other exclusion criteria were death < 4 weeks of life, babies prenatally exposed to anticonvulsants and enteral calcitriol, and who needed dialysis in the perinatal period. All the neonates included in the final cohort were further subdivided into the high ALP and low ALP groups based on the serum-ALP levels at the 4^th^ week of life.

### Data collection

Information was extracted from hospital electronic health records. This included information on prenatal and postnatal clinical characteristics including gender, GA, and BW. Data information about the caffeine regimen included daily doses (mg/kg/day), cumulative doses (mg), and duration of caffeine therapy. In addition, information was retrieved about other concomitant risk factors for OOP including average weekly Ca-intake (mg/kg), P-intake (mg/kg), vitamins-D-intake (IU), and exposure to steroids (cumulative doses in mg/kg) and diuretics (cumulative doses in mg/kg). Information was retrieved about the duration of phenobarbital, paralytic agents, and parenteral nutrition (PN) during 4 weeks of neonatal life. Duration of PN counts for total PN and partial PN days, as in the study center PN is continued till > 100 ml/kg/day EN tolerance (supplementary file). Laboratory parameters included serum-ALP and serum-P at the 4^th^ week of life.

In this retrospective study, the serum-ALP concentration at the 4^th^ week of life was used as a surrogate marker for the risk of developing OOP in preterm neonates as radiographic studies were not performed on all neonates till the 4^th^ week of life for evaluating the risk of osteopenia [20, 21, 23]. Reported cutoff values of serum-ALP concentration of 500 IU/L were used, at which osteopenia is detected with 100% sensitivity and 80.77% specificity [21]. The serum-P levels were recorded for all the patients. Neonates with serum-P levels lower than 5.6 mg/dL (< 1.8 mmol/L) are reported to have a greater risk for MBD and development of OOP with diagnostic sensitivity and specificity of 70% and 100%, respectively [20].

### Statistical analysis

Descriptive analysis was undertaken using STATA version 17 (Stata Corp, Texas) for all study variables stratified into high ALP and low ALP groups. Categorical variables were analyzed by applying the χ2 test or Fisher exact test, as appropriate. For continuous variables applied the Student t-test or Wilcoxon Mann–Whitney median tests, as appropriate. Data were summarized using number (%) for categorical variables and mean ± SD or median (IQR) for continuous variables as appropriate. The predictors of OOP were determined by multivariable logistic regression after an initial univariable analysis. Variables significant at *p* < 0.2 in the univariable analysis were included in the fully adjusted model. The final model was constructed using backward elimination, variables being retained if *p* < 0.05. A *p*-value less than 0.05 was taken as significant. The probability of OOP by GA was evaluated.

### Ethical approval and consent to participate

Before performing this study, ethical approval was taken from the institutional ethical committee of Aga Khan University Hospital (ERC # 2019-2111-5600) and informed consent from a parent or guardian for participants was waived by ERC as all the data was collected retrospectively.

## Results

### Patient demographics and serum ALP levels

**Fig. 1:**
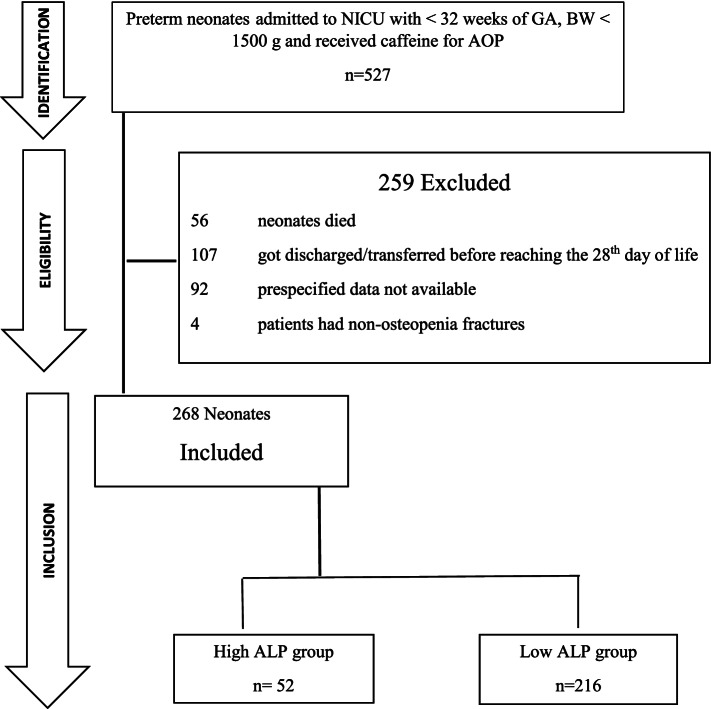
Flow chart depicting recruitment of cohort

A total of 527 preterm neonates were included in the initial cohort, with GA < 32 weeks and BW < 1500 g, who were admitted to NICU and administered caffeine for treating AOP during the study period. A flow chart depicting the recruitment of the final cohort shows the detail (Fig. 1). The final study group included 268 neonates who had at least 28 days of hospital stay, clinical information, and laboratory data for analysis.

**Fig. 2:**
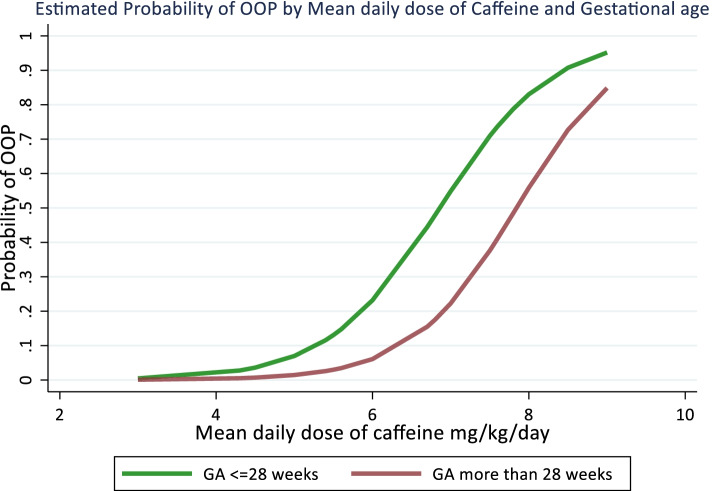
Probability of osteopenia of prematurity (OOP) with increasing mean caffeine daily dose at GA ≤ 28 weeks and > 28 weeks

**Fig. 3:**
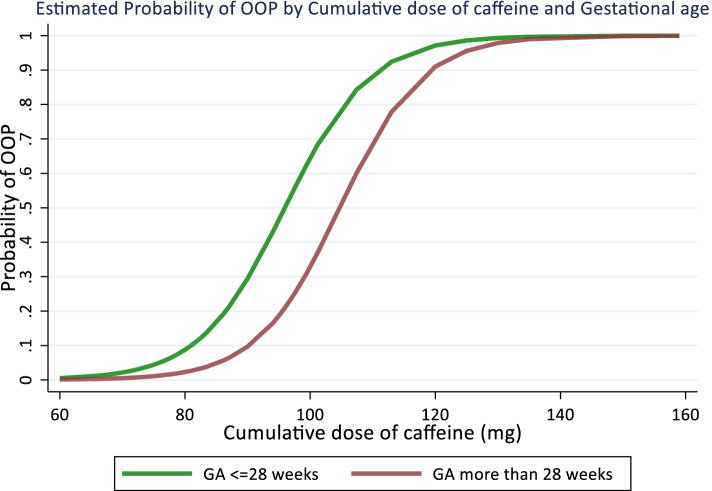
Probability of osteopenia of prematurity (OOP) with increasing cumulative caffeine dose at GA ≤ 28 weeks and > 28 weeks

Baseline demographics and laboratory information of the high ALP and low ALP groups are shown in Table 1. Of the total of 268 participants, 52 (19%) developed OOP, and a significantly (*p* = 0.027) higher frequency was observed in female patients. In the high ALP group, the serum-ALP levels at 28 weeks were significantly higher than in the low ALP groups (725.0 ± 143.8 vs 273.6 ± 55.0 units/L, *p* < 0.001). The serum-P levels were significantly lower in high ALP (2.6 ± 0.5 vs 5.7 ± 0.6 mg/dl, *p* < 0.001). The high ALP was observed to be more premature and had a longer duration of NICU stay till the 28^th^ day of life. The probability of OOP was higher in neonates of GA ≤ 28 weeks than the neonates of GA > 28 weeks with increasing mean daily dose and cumulative dose of caffeine (Figs. 2&3). Male children have significantly higher median GA as compared to females (29 vs 28 weeks, *p* < 0.001).

**Table 1:** Demographic And Laboratory Characteristics

Variables	High ALP group (*N* = 52)	Low ALP group (*N* = 216)	*p*-value
**Gender** ^#^			
Male	20 (38.5%)	120 (55.6%)	0.027
Female	32 (61.5%)	96 (44.4%)	
**GA (weeks)** ^@^	28.0 (27.0–28.0)	29.0 (28.0–31.0)	< 0.001
**BW (gm)** ^@^	955.0 (800.0–1400.0)	1200.0 (850.0–1420.0)	0.026
**Length of NICU stay (days)**^**+**^	28.0 (21.0–40.0)	21.0 (15.0–26.0)	< 0.001
**Maternal parity level** ^#^			
Low < 2	34 (65.4%)	122 (56.5%)	0.10
Moderate 2–4	8 (15.4%)	64 (29.6%)	
High > 4	10 (19.2%)	30 (13.9%)	
**Serum ALP levels (units/L)**			
**Mean** ± **SD**	725.0 ± 143.8	273.6 ± 55.0	< 0.001
≤ 500 ^#^	0 (0.0%)	216 (100.0%)	< 0.001
> 500 ^#^	52 (100.0%)	0 (0.0%)	
**Serum phosphorus levels (mg/dl) **^**##**^	2.6 ± 0.5	5.7 ± 0.6	< 0.001

Data presented as ^#^ number (%), ^##^ Mean ± SD and ^@^ median (range) values. *ALP* Alkaline Phosphatase, *GA* Gestational age, *BW* Birth weight, *NICU* Neonatal intensive care unit. + From total of 28 days of hospital stay

### Caffeine regimen

The caffeine regimen of the study cohort is summarized in Table 2. Overall, neonates in the high ALP group received a significantly (*p* < 0.001) higher cumulative caffeine dose in 28 days of therapy (median 110.2 mg, range 96.0–125.0 mg). The high ALP group received a median daily dose of 10.0 mg/kg (range 7.5–10.0 mg), which was significantly (*p* < 0.001) higher than the low ALP group neonates, who received a median daily dose of 5.5 mg/kg (range 5.0–7.0 mg).

**Table 2:** Caffeine therapy of neonates in OOP and non-OOP groups

Variables	High ALP group (N = 52)	Low ALP group (N = 216)	*p*-value
Duration of therapy of caffeine (days) ^##^	28.0 ± 0.0	27.7 ± 0.8	0.008
Cumulative dose of caffeine (mg) ^@^	110.2 (96.0–125.0)	74.8 (70.0–80.0)	< 0.001
Maximum daily dose of caffeine (mg/kg/day) ^@^	10.0 (7.5–10.0)	5.5 (5.0–7.0)	< 0.001

Data presented as ## Mean ± SD and @ median (range) values. *ALP* Alkaline Phosphatase

### Other risk factors associated with developing OOP

Other risk factors associated with developing OOP in neonates are compared in Table 3. Infants in both groups got exposure to other medications which have the potential to develop osteopenia. A substantial number of infants in both the groups were exposed to a loop diuretic (i.e., furosemide) but statistically, no significant difference was observed. Both the groups were statistically not different for the exposure and total duration of paralytic agents. The use of phenobarbital was also comparable in both groups. Similarly, no significant difference was observed in usage and cumulative dose of steroids between the groups. Groups were compared for steroid exposure after converting steroid equivalents (i.e., each 20 mg hydrocortisone dose to 0.75 mg dexamethasone). Infants in both groups received parenteral nutrition (PN) for the more or less same duration (*p* = 0.69).

**Table 3:** Neonatal Clinical Data for Concomitant Risk Factors Associated with Developing Osteopenia

Variables	High ALP group (N = 52)	Low ALP group (N = 216)	*p*-value
**Duration of PN (days)** ^##^	8.8 ± 5.4	8.5 ± 5.2	0.69
**Used steroids** ^#^			
Yes	14 (26.9%)	56 (25.9%)	0.88
No	38 (73.1%)	160 (74.1%)	
**Used diuretics** ^#^			
Yes	36 (69.2%)	166 (76.9%)	0.25
No	16 (30.8%)	50 (23.1%)	
**Used Phenobarbital** ^#^			
Yes	8 (15.4%)	40 (18.5%)	0.60
No	44 (84.6%)	176 (81.5%)	
**Used Paralytic agent** ^#^			
Yes	4 (7.7%)	10 (4.6%)	0.37
No	48 (92.3%)	206 (95.4%)	
**Cumulative steroid dose (mg/kg) **^**##**^	1.1 ± 0.9	1.0 ± 1.1	0.78
**Days of paralytic drug **^**##**^	1.0 ± 0.0	1.0 ± 0.0	-

Data presented as # number (%), ## Mean ± SD and @ median (range) values. *ALP* Alkaline Phosphatase, *PN* Parenteral Nutrition

Groups were further evaluated for the intake of vitamins and essential minerals needed for bone growth through both PN and enteral nutrition (EN). Statistically significant difference was observed in P-intake (mg/kg) at week 1 (*p* = 0.027), week 2 (*p* = 0.023), and week 4 (*p* = 0.032). However, data analysis revealed no significant difference in calcium (mg/kg) and vitamin D (IU) intake for four weeks (Table 4).

**Table 4:** Weekly Comparison Between the Groups for Phosphorus, Calcium and Vitamin D Intake

Variables	High ALP group (N = 52)	Low ALP group (N = 216)	*p*-value
**Mean Phosphorus intake** (mg/kg) ^##^			
week 1	40.8 ± 13.4	45.6 ± 14.0	0.027
week 2	47.7 ± 21.9	50.3 ± 23.0	0.023
week 3	60.7 ± 23.5	63.8 ± 21.5	0.12
week 4	74.7 ± 22.0	78.3 ± 26.8	0.032
**Mean Calcium intake** (mg/kg) ^##^			
week 1	162.4 ± 91.2	164.9 ± 93.9	0.86
week 2	210.4 ± 81.5	212.9 ± 74.5	0.86
week 3	237.4 ± 75.9	239.9 ± 81.2	0.86
week 4	210.4 ± 28.9	212.9 ± 90.8	0.86
**Mean Vitamin D intake** (IU) ^##^			
week 1	544.7 ± 85.3	548.3 ± 78.0	0.77
week 2	557.7 ± 85.0	561.3 ± 75.1	0.77
week 3	570.2 ± 72.9	569.1 ± 79.9	0.93
week 4	557.7 ± 81.5	558.0 ± 82.9	0.98

Data presented as ## Mean ± SD. *ALP* Alkaline Phosphatase

### Regression analysis

Table 5 describes the predictors associated with OOP by multivariable logistic regression after initial univariable analysis. The GA and BW were inversely related to the occurrence of OOP in univariate analysis. Neonates of smaller GA were more likely to develop OOP (AOR = 0.244 95% CI 0.068 to 0.876) in multivariate analysis. Female participants were found at higher risk for OOP (AOR = 0.695 95% CI 0.483 to 0.999). Moderate maternal parity was significantly associated with OOP in univariable analysis but reported insignificant association in multivariate analysis. The higher cumulative dose of caffeine (mg) (AOR = 1.082 95% CI 1.011 to 1.157) and a higher daily dose of caffeine (mg/kg/day) (AOR = 2.892 95% CI 1.392 to 6.007) in 28 days of therapy were associated with a higher likelihood of developing OOP in this study cohort. The intake of minerals and vitamins had no association with the development of OOP in bivariate analysis, however, per unit (mg/kg) increase in phosphorus intake was associated with a reduction in OOP. This effect is significant only at week 1 (crude OR = 0.976, *p* = 0.027), week 2 (crude OR = 0.999, *p* = 0.023) and week 4 (crude OR = 0.999, *p* = 0.032). In adjusted analysis phosphorus intake was not associated with OOP.

**Table 5:** Risk Factors Associated With OOP: Results of Univariate and Multivariable Analysis

Variables	OOP		Crude OR (95% CI)	*p*-value	Adjusted OR (95% CI)	*p*-value
	**No (N = 216)**	**Yes (N = 52)**				
**Gender **^#^						
Male	120 (55.6%)	20 (38.5%)	Ref	Ref	Ref	Ref
Female	96 (44.4%)	32 (61.5%)	2 (1.076—3.717)	0.028	0.244 (0.068—0.876)	0.030
GA (weeks) @	29.0 (28.0–31.0)	28.0 (27.0–28.0)	0.656 (0.532—0.808)	< 0.001	0.695 (0.483—0.999)	0.050
BW (gm) @	1200.0 (850.0–1420.0)	955.0 (800.0–1400.0)	0.999 (0.998—1)	0.011	-	-
**Maternal parity level** ^#^						
Low < 2	122 (56.5%)	34 (65.4%)	Ref	Ref	-	-
Moderate 2–4	64 (29.6%)	8 (15.4%)	0.449 (0.196—1.026)	0.048	-	-
High > 4	30 (13.9%)	10 (19.2%)	1.196 (0.532—2.69)	0.665	-	-
**Caffeine therapy**						
Duration of therapy (days) ##	27.7 ± 0.8	28.0 ± 0.0	-	-	-	-
Cumulative dose (mg) @	74.8 (70.0–80.0)	110.2 (96.0–125.0)	1.159 (1.112—1.209)	< 0.001	1.082 (1.011—1.157)	0.022
Maximum daily dose (mg/kg/day) @	5.5 (5.0–7.0)	10.0 (7.5–10.0)	5.668 (3.27—9.826)	< 0.001	2.892 (1.392—6.007)	0.004
**Concomitant therapy and risk factors of Osteopenia**						
Duration of PN (days) ^##^	8.5 ± 5.2	8.8 ± 5.4	1.012 (0.955—1.071)	0.692	-	-
used steroids ^#^						
No	160 (74.1%)	38 (73.1%)	Ref	Ref	-	-
Yes	56 (25.9%)	14 (26.9%)	1.053 (0.531—2.086)	0.883	-	-
used diuretics ^#^						
No	50 (23.1%)	16 (30.8%)	Ref	Ref	-	-
Yes	166 (76.9%)	36 (69.2%)	0.678 (0.347—1.322)	0.254	-	-
used Phenobarbital ^#^						
No	176 (81.5%)	44 (84.6%)	Ref	Ref	-	-
Yes	40 (18.5%)	8 (15.4%)	0.8 (0.35—1.831)	0.597	-	-
used Paralytic agent ^#^						
No	206 (95.4%)	48 (92.3%)	Ref	Ref	-	-
Yes	10 (4.6%)	4 (7.7%)	1.717 (0.516—5.707)	0.378	-	-
Cumulative steroid dose (mg/kg) ^##^	1.0 ± 1.1	1.1 ± 0.9	1.08 (0.633—1.844)	0.777	-	-
Days of paralytic drug ^##^	1.0 ± 0.0	1.0 ± 0.0	-	-	-	-
**Comparison of mean phosphorous intake (mg/kg)** ^##^						
week 1	45.6 ± 14.0	40.8 ± 13.4	0.976 (0.955—0.998)	0.027	-	-
week 2	50.3 ± 23.0	47.7 ± 21.9	0.999 (0.986—1.012)	0.023	-	-
week 3	63.8 ± 21.5	60.7 ± 23.5	0.998 (0.985—1.011)	0.12	-	-
week 4	78.3 ± 26.8	74.7 ± 22.0	0.999 (0.986—1.012)	0.032	-	-
**Comparison of mean calcium intake (mg/kg)** ^##^						
week 1	164.9 ± 93.9	162.4 ± 91.2	1 (0.996—1.003)	0.864	-	-
week 2	212.9 ± 93.9	210.4 ± 91.2	1 (0.996—1.003)	0.864	-	-
week 3	239.9 ± 93.9	237.4 ± 91.2	1 (0.996—1.003)	0.864	-	-
week 4	212.9 ± 93.9	210.4 ± 91.2	1 (0.996—1.003)	0.864	-	-
**Comparison of mean vitamin D intake (IU)** ^##^						
week 1	548.3 ± 78.0	544.7 ± 85.3	0.999 (0.996—1.003)	0.772	-	-
week 2	561.3 ± 78.0	557.7 ± 85.3	0.999 (0.996—1.003)	0.772	-	-
week 3	569.1 ± 79.9	570.2 ± 72.9	1 (0.996—1.004)	0.927	-	-
week 4	558.0 ± 82.9	557.7 ± 85.3	1 (0.996—1.004)	0.981	-	-

^#^ Data presented as # number (%), ## Mean ± SD and @ median (range) values. *OOP* Osteopenia of prematurity, *GA* Gestational age, *BW* Birth weight, *NICU *Neonatal intensive care unit, *ALP* Alkaline phosphate

## Discussion

Our study reported the association between caffeine dose and the presence of osteopenia of prematurity (OOP) in preterm neonates. The incidence of OOP varies among neonatal studies depending upon the population, sample size, study design, or serum-ALP and serum-P levels threshold applied for detecting OOP. Caffeine consumption has been recognized as a risk factor for low BMD and fractures in postmenopausal and adolescent females [24–26]. This is considered to be the result of caffeine-induced increased intestinal and urinary calcium excretion [26]. Neonatal studies also reported the renal effects of caffeine, including diuresis, hypercalciuria, and natriuresis [15, 16, 27].

In addition to the known caffeine’s beneficial long-term outcomes for treating AOP [28], our results revealed an association between daily and cumulative dosages of caffeine and the risk of developing OOP even with the control of other risk factors. Similar results are reported by Ali E et al. but they also related prolonged duration of caffeine therapy with OOP [29]. However, in our study neonates received caffeine therapy for 28 days in both groups. Similarly, another recent study only tracked the duration of caffeine therapy and reported no difference between the control and OOP groups. [30]. There have been many RCTs using higher doses of caffeine, a recent one reported the effects on the development of OOP and stated no significant difference between the high and low dose groups [31]. However, the doses used in the intervention and control groups were very higher than in our study, and the neonates with comparatively bigger GA were included [31].

Likewise, in our study neonates of lower GA were at a higher risk of developing OOP with caffeine therapy [29, 32]. That might be explained by the prolonged caffeine half-life due to compromised renal functions and slow metabolism due to immature hepatic enzymes leading to sustained concentration causing calciuria that stimulates the release of parathyroid hormone (PTH), an ultimate trigger for an increased osteoclastic activity for releasing calcium from the bone matrix and osteoclastogenesis [33, 34]. It is possible that sicker and smaller neonates have more AOP and get more caffeine and therefore are at a higher risk to develop OOP [5].

In this study cohort, OOP was higher in female infants, these results are comparable to previous neonatal studies. They also found higher bone density in male infants than in females. Such an observation may follow a recognizable trend for testosterone hormone in utero [35, 36]. This higher rate of OOP in females might be related to survival bias as female participants in this study cohort have significantly smaller median GA as compared to males. However, few studies reported no difference in the incidence of OOP concerning gender [29, 32].

All the 52 neonates who developed OOP received higher daily doses of caffeine [median 10.0 mg/kg/day (range 7.5–10.0 mg/kg/day)]. The higher daily dose was used and titrated upward in neonates who persistently had apnea, considering tachycardia as the dose-limiting factor. On the other hand, all the neonates who did not develop OOP received lower daily doses of caffeine [5.5 mg/kg/day (range 5.0–7.0 mg/kg/day)]. Similar effects of high dose caffeine are reported in a recent study [29], however, in contrast to our results Miller JL et al. reported no significant difference in ALP maximum concentration between low and high dose groups [32].

This study showed no difference in the duration of PN therapy among the neonates who developed OOP and who were safe. Our results are comparable with Ali E et al. study, as they also found no association between duration of PN and OOP development when controlled for other risk factors [29]. These results might be justified by considering the fact that PN compounded with the highest amount of phosphate and calcium with the highest solubility allowed [37] and according to individual patient serum levels and needs.

Although in this study ALP > 500 units/L was considered as the surrogate marker of increased risk of OOP in neonates, however, we found considerably lower serum-P concentrations at the fourth week of life in the high ALP group. In our study, all the neonates who had ALP < 500 units/L reported serum-P concentration > 5.1 mg/dl. Our results are supported by Backström et al., who suggested OOP diagnostic sensitivity of 100% and specificity of 70% with serum-P concentration < 5.5 mg/dl (1.8 mmol/L) [22]. In agreement with our results, few other studies reported a correlation of serum-P and serum-ALP levels with the OOP development in late infancy, which could be explained by the other confounding factors and medications received that affects premature bone in early life in NICUs [38, 39]. However, Ali E et al. measured serum-P concentrations on a biweekly basis and reported no significant relation with OOP. Though serum-P is one of the tightly regulated minerals, average single or biweekly readings might not be the true representative of the real situation of serum-P in infants on total or partial PN for the first few weeks of their lives. Likewise, Aly et al., (2005) reported no correlation between serum-P single record at birth with OOP in preterm neonates [36].

Our study evaluated the concomitant risk factors for the development of OOP and found a significant difference between groups in the intake of phosphorus. Neonates at the risk of developing OOP received a lesser amount of phosphorus on weeks 1, 2, and 4. Although intake of calcium and vitamin D was comparable in both groups. In the interpretation of these findings, it is important to mention that 35/52 neonates in the high ALP group received exclusively mother milk when switched to EN. However, in the low ALP group neonates received premature formula feed as well, although the duration of exclusive PN was comparable between the groups. Nevertheless, the small number of cases in the high ALP group and the uneven distribution between groups may have influenced the results.

Our study reports no difference between the use of furosemide and steroids in both the groups and no effect on the higher risk of OOP. These results might be explained by the lower cumulative doses of steroids and diuretics in this study cohort. The lower need for diuretics might be explained by the diuretic effect of caffeine and lesser use of steroids in our unit for neonates less than four weeks of life.

Higher maternal parity was reported to have a negative correlation with the mother’s BMD measurements but no significant effects on the baby’s bone formation. This establishes the statement that a fetus gets the required vitamins and minerals from the mother via concentration gradient-driven active transport passing over the mother’s state [40]. In this cohort, we found no significant effects of maternal parity on OOP. Similar findings are reported by a previous study [29]. However, further studies with enough high parity mothers can better identify this correlation, as in our study cohort the number was not enough.

There are a few inherent limitations involved in this study due to its retrospective nature. Raised serum-ALP was selected as a biochemical marker for the risk of developing OOP, as reported to have a positive correlation [41, 42]. However, the suitability of serum-ALP as a surrogate biochemical marker of osteopenia has been debated [43], and radiographic findings are typically considered for the diagnosis of osteopenia. Though, in this retrospective study design, radiographic examination for OOP was not performed for any case till the 4^th^ week of life. Neonates were excluded from the initial study group based on incomplete information about the serum-ALP and serum-P levels. The influence of multiple risk factors for developing OOP is another limitation of this study. However, an attempt was made to address this issue by collecting information on these known factors and analyzing the results in this context. But, in this retrospective study, we could not get documented information on mothers' intake of caffeine through pregnancy and lactation period, which can negatively affect bone formation and development [44]. The smaller number of neonates in the high ALP group is an additional limitation, who were at a higher risk of developing OOP with elevated serum-ALP levels and lower serum-P levels. Therefore, to overcome this type-II error in reporting results, a linear regression was utilized to identify factors with significant relationships to the risk of developing OOP in neonates who reported raised serum-ALP levels.

## Supplementary Information

**Additional file 1.**

## Data Availability

All data generated or analyzed during this study are included in this published article. The datasets used and/or analyzed during the current study are available from the corresponding author on reasonable request.

## Abbreviations

NICU: Neonatal Intensive Care Units
AOP: Apnea of Prematurity
MBD: Metabolic Bone Disease
BR: Bone Resorption
OOP: Osteopenia of Prematurity
Serum-ALP: Serum Alkaline phosphatase
Serum-P: Serum Phosphorous
Serum-Ca levels: Serum Calcium levels
GA: Gestational Age
PMA: Postmenstrual Age
BW: Birth Weight
PN: Parenteral Nutrition
EN: Enteral Nutrition
