# Supplementary material for: Relationship of caffeine regimen with osteopenia of prematurity in preterm neonates: a cohort retrospective study
Source: BMC Pediatr. 2022 Jul 21;22:437. doi: 10.1186/s12887-022-03493-x (PMC9306044; doi:10.1186/s12887-022-03493-x)
Supplement: Supplementary file 1 — Additional file 1. [file 12887_2022_3493_MOESM1_ESM.pdf]

Supplementary Material, Part1: ENTERAL FEEDING PROTOCOL

Introducing and advancing neonatal feeds

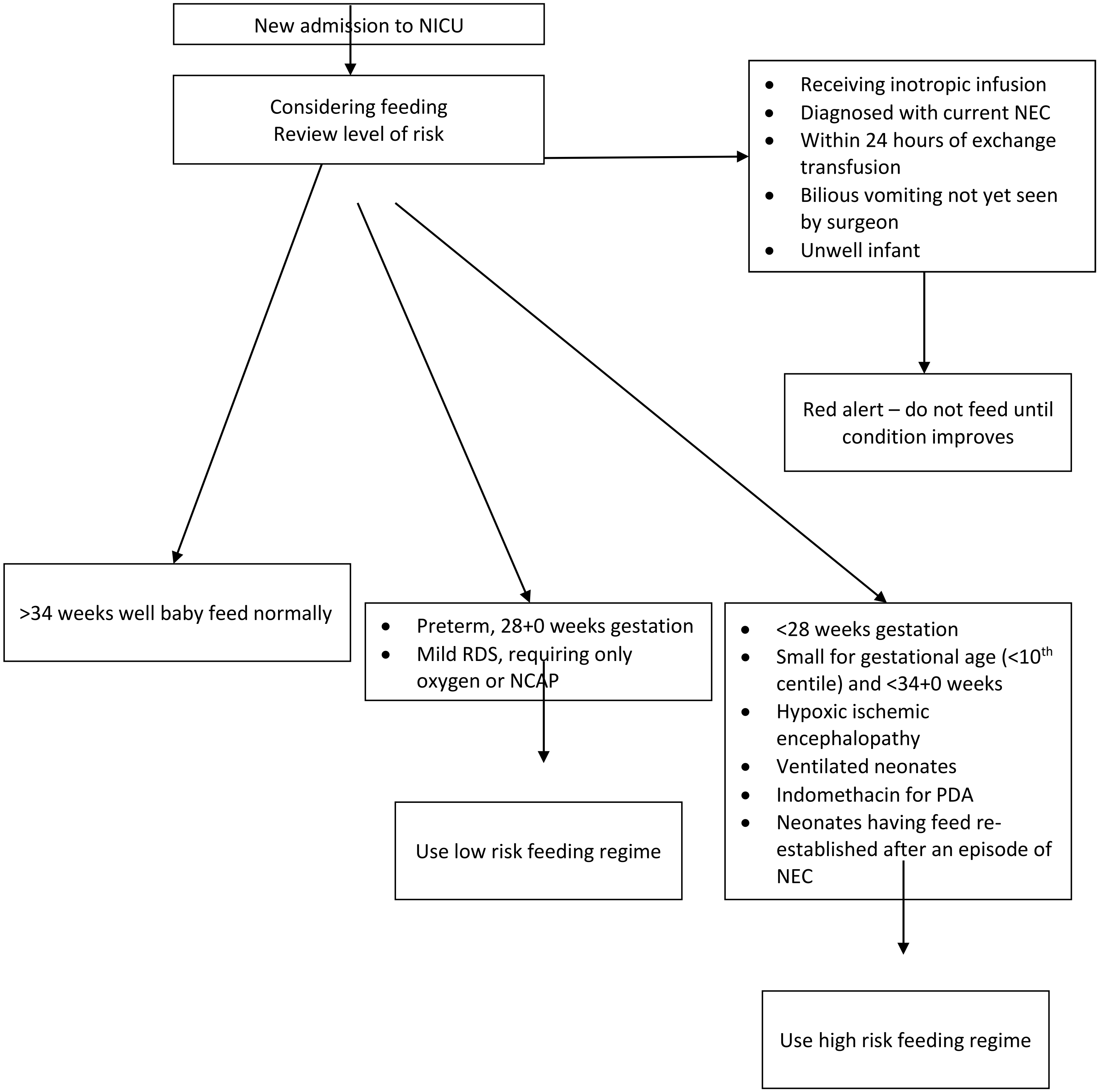

**Starting feeds**: Infants born after 34 weeks of gestation and weighing more than 1.5kg (irrespective of IUGR or antenatal Doppler) can be fed immediately provided they are well. Full feeds can be established as soon as the infant is able to tolerate either orally or via NG tube.		
	**Patient condition**	**First feed advice**
Low risk infants	32+0 and above	May tolerate full feeds immediately: so trial these at 40ml/kg/day using 2, 3 hourly feeds
	30+0 - 31+6 weeks inclusive and well	Start 2 hourly bolus as soon as possible after birth, at step 2
	28+0 - 29+6 weeks inclusive and well	Start feeds in first 24 hours and review every 24 hours, at step 1
High risk infants	23+0 – 27+6 weeks and well or another risk factor (see summary)	Consider starting trophic feeds** in first 24 hours and review carefully every 24 hours, increasing according to tolerance, proceed to step 1 after 24 hours
	<34 weeks and AREDF* or small for gestational age (<10th centile)	
Red alert	Active NEC diagnosed<br>Exchange transfusion in the last 24 hours<br>Bilious vomiting not seen by surgeon<br>Unwell infant	Withhold feeds – NPO until condition improves

*ARDEF=Absent or reduced end diastolic flow

**Trophic feeds=0.5-1.5ml per feed 6 hourly

**Advancing feeds**			
**Step**	**Feed volume 3 hourly**	**Increment**	**End of day rate**
Low risk infants			
1	1ml/kg	24 hours	8ml/kg/24 hours
2	3ml/kg<br>4ml/kg	12 hours<br>12 hours	28ml/kg/24 hours (an increase of 20ml/kg/day)
3	8ml/kg<br>10ml/kg<br>12ml/kg	8 hours<br>8 hours<br>8 hours	60ml/kg/24 hours (an increase of 32ml/kg/day)
4	15ml/kg<br>18ml/kg<br>20ml/kg	8 hours<br>8 hours<br>8 hours	100ml/kg/24 hours (an increase of 40ml/kg/day)
5	At attending neonatologist’s discretion		
High risk infants			
1	1ml/kg	24 hours	8ml/kg/24 hours

2	3ml/kg<br>4ml/kg	12 hours<br>12 hours	28ml/kg/24 hours (an increase of 20ml/kg/day)
3	5ml/kg<br>6ml/kg	12 hours<br>12 hours	52ml/kg/24 hours (an increase of 24ml/kg/day)
4	8ml/kg<br>10ml/kg	12 hours<br>12 hours	72ml/kg/24 hours (an increase of 20ml/kg/day)
5	12ml/kg<br>14ml/kg	12 hours<br>12 hours	104ml/kg/24 hours (an increase of 32ml/kg/day)
6	16ml/kg<br>18ml/kg	12 hours<br>12 hours	136ml/kg/24 hours (an increase of 32ml/kg/day)
7	19ml/kg<br>20ml/kg	12 hours<br>12 hours	156ml/kg/24 hours (an increase of 20ml/kg/day)
8	At attending neonatologist's discretion		

<u>Supplementary Material, Part2: Enfamil® Premature 20</u>

Enfamil® Premature 20

Nutrient	Per 100 Cal	Per Liter
**Energy, Cal**	**100**	**676**
**Volume, mL**	**148**	**1000**
**Protein**, g	3.3	22
% of total calories†	13	13
**Fat**, g	5	34
% of total calories††	44	44
Linoleic acid, mg	810	5500
**Carbohydrate**, g	10.8	73
% of total calories§	43	43
**Water**, g	132	890
**Minerals**		
Calcium, mg (mEq)	165 (8.2)	1120 (56)
Phosphorus, mg (mEq)	90 (2.9)	610 (19.7)
Magnesium, mg	9	61
Iron, mg	1.8*	12.2*
Zinc, mg	1.5	10.1
Manganese, mcg	6.3	43
Copper, mcg	120	810
Molybdenum, mcg	-	-
Iodine, mcg	25	169
Selenium, mcg	5	34
Sodium, mg (mEq)	70 (3)	470 (20.4)
Potassium, mg (mEq)	98 (2.5)	660 (16.9)
Chloride, mg (mEq)	106 (3)	720 (20.3)
**Vitamins**		
Vitamin A, IU	1350	9100
Vitamin D, IU	300	2000
Vitamin E, IU	6.3	43
Vitamin K, mcg	9	61
Thiamine (B_1), mcg	200	1350
Riboflavin (B_2), mcg	300	2000
Vitamin B_6, mcg	150	1010
Vitamin B_{12}, mcg	0.25	1.7
Niacin, mcg	4000	27,000
Folic acid (Folacin), mcg	40	270
Pantothenic acid, mcg	1200	8100
Biotin, mcg	4	27
Vitamin C (Ascorbic acid), mg	20	135
Choline, mg	24	162
Inositol, mg	44	300
L-Carnitine, mg	--	--
Taurine, mg	--	--
Nucleotide fortification, mg	-	-
**Renal Solute Load**, mOsm	30	200
**Osmolality**, mOsm/kg H2O	260	260

**Osmolarity**, mOsm/L	230	230

20 Cal/fl oz
Available as ready-to-feed.
Intake of some nutrients such as fat-soluble vitamins may be excessive when more than 14 fl oz (414 mL) of 20 cal/fl oz formula is consumed per day; such use should be only under medical supervision.

Iron supplementation should be considered for premature infants.

† Protein Source: Nonfat milk and whey protein concentrate (80% whey, 20% casein).

†† Fat Source: Medium-chain triglyceride oil (40%), soy oil, high oleic sunflower oil, and single-cell oil blend rich in docosahexaenoic acid and arachidonic acid (less than 0.5%).

§ Carbohydrate Source: Corn syrup solids (60%) and lactose (40%).

Supplementary Material, Part3: TPN

Composition of TPN

Constituents Per 100ml	TPN (each 100ml)
Amino acids (gm)*	2.5
Dextrose(gm)	8-10
Lipid (gm) (20% IVFE)#	0-1.9
Magnesium(mEq)	0.4
Potassium(mEq)	0-1.5
Phosphorus(mmol)**##	0-1
Sodium Chloride(mEq)	2.5-3
Zinc (mcg)	300
Calcium-Gluconate(mg)##	125
Multivitamin(1ml)	1
Heparin (IU)	50

IVFE= Intravenous Fat Emulsion; *Aminoplasmal® B. Braun 10%, ** potassium phosphate salt, # 20% Lipofundin ® B. Braun,

Nutrition protocol for preterm neonates who received TPN formula
Increment of TPN (ml/kg/day)

TPN volume	Daily values
**TPN volume (ml/kg/day)**	Day 1: 80–100<br>Day 2: 110–120<br>Day 3: 120–140<br>Day 4 & 5: 140–160<br>From day 6: maximum of 160–180 to 200 depending on the neonate's birth weight and clinical condition
